# Unfolding alignment – How top management work to align demand and capacity: an ethnographic study of resilience in a Swedish healthcare region

**DOI:** 10.1186/s12913-023-09291-0

**Published:** 2023-03-31

**Authors:** Ingrid Svensson, Mia von Knorring, Heidi Hagerman, Cecilia Fagerström, Mirjam Ekstedt, Lisa Smeds Alenius

**Affiliations:** 1 Department of Learning, Informatics, Management and Ethics, LIME, Karolinska Institutet, Tomtebodavägen 18 A, SE-17165 Solna, Sweden; 2 Faculty of Health and Life Sciences, Dept of Health and Caring Sciences, Kalmar/Växjö Linnaeus University, Universitetsplatsen 1, 392 31 Kalmar, Sweden

**Keywords:** Alignment, CARE-model, Ethnographic study, Healthcare, Resilience, Top management, Leadership, Thematic analysis

## Abstract

**Background:**

Resilient healthcare organizations maintain critical functions and high-quality care under varying conditions. While previous research has focused on the activities of frontline healthcare professionals working at the “sharp end” of care, less attention has been paid to managers at the top management level. More knowledge is needed to fully understand how the managers align demand and capacity at the “blunt end” of care. Therefore, this study aimed to explore how top managers work to align demand and capacity in a healthcare region in Sweden.

**Methods:**

Observations of management team meetings, interviews, and conversations were conducted with top managers responsible for healthcare in one of Sweden’s 21 regions. Data collection used an ethnographic approach. Data were analyzed using qualitative reflexive thematic analysis.

**Results:**

The data showed how alignment work was done through active reflection that built on past experiences and on structures built into the organization at the same time as taking future potential outcomes and consequences into account. In addition to collaborative, preventive, supportive, and contextualizing work, which was conducted in the present, a general approach permeated the organization, which enabled connecting actions, i.e., different forms of alignment work, occurring at different points in time, and connecting different types of knowledge across organizational borders and stakeholders.

**Conclusion:**

This study explored how top managers work to align demand and capacity in a healthcare region in Sweden. It was shown how four categories of work; collaborative, preventive, supportive and contextualization work, together with a general approach; focusing on opportunities, building on a stable past and taking a reflective stance, constitute alignment in practice. More; the alignment work was done in the here and now, with both the past and future in mind. The ability to take action to benefit the whole is a possibility and a responsibility for top management. In the region studied, this was done by aligning demands with capacity based on past experiences and focusing on the available opportunities to connect knowledge needed within and across organizational borders.

**Supplementary Information:**

The online version contains supplementary material available at 10.1186/s12913-023-09291-0.

## Background

Resilient healthcare organizations are characterized by their ability to maintain critical functions serving to deliver safe high-quality care under varying conditions [1, 2]. Since outcomes at the patient level are influenced by actions at multiple system levels, not just by the staff providing direct bedside care, it is important to explore expressions of resilience activities at the top management levels to more fully understand the mechanisms affecting patient care in the long term [3–5]. However, to date, most research on resilience in healthcare has focused on activities at the “sharp end” of care [6–8], e.g., how frontline healthcare professionals adapt, or apply work-arounds, to deal with mis-alignments [4, 9].

A common strategy for studying resilience processes in organizations is to use the cornerstones of resilience i) *anticipating* [2, 10, 11], ii) *monitoring* [2, 10], iii) *responding* [2, 10], iv) *learning* [2, 10, 12], v) *coordinating* [10, 12], vi) *sense-making* [11], vii) *trade-offs* [11], and viii) *adaptions* [11]. These cornerstones are seen as interdependent activities and highlight the importance of being aware of context, using fore- and hindsight, and making sense and learning from both expected and unexpected events.

In a study exploring resilience activities from the perspective of adaptive capacity for resilience, Lyng et al. [3] found examples of adaptive capacity across different healthcare contexts, taking the following forms: reframing practices, aligning different perspectives, coping with demands, and innovating. These adaptive capacities in turn are influenced by knowledge, communication, organizational resources, and trust and are applied in response to external and internal demands from different organizational levels, to ensure quality of care [3].

The different activities and capacities presented in previous research are described to be present across system levels and organizations [3, 11], and will usually involve the actions of a wide range of actors across several organizational levels of healthcare (e.g., managers, healthcare workers, patients, stakeholders, and policymakers etc.) [4].

To operationalize and visualize the resilient system approach to quality improvement Andersson et al. [13] developed the Concepts for Applying Resilience Engineering (CARE) model. The model takes into account the different layers of the healthcare system and acknowledges that quality problems are emergent properties of system complexity [13]. It was created as a clinical practice tool to guide quality improvement in front-end work [10]. Inherent to the CARE model is the idea that the healthcare system must be designed to support resilience activities in practice [13]. Here, it is the system’s ability to perform that is of interest and importance, not the abilities of individual actors. Part of designing the system and creating pre-conditions for resilience is done in the realm of what in the CARE model is called “work-as-imagined”. In this realm guidelines and procedures are created by (top)management and policymakers with the (implicit) aim of aligning *demand* and *capacity*. Demand, in the CARE model, is described as something which “generate[s] things that need to be done” [10] whereas capacity refers to the resources available to meet those demands. *Alignment,* situated between demand and capacity, is described as “the organization’s best guess of what is required day to day, based on previous experience and anticipated demand” [10]. As a result of the orientation towards use in clinical practice, examples of what is included in *demand* and *capacity* focus on the sharp end perspective (e.g., demand includes patient numbers and seasonal changes, whereas capacity includes staffing levels, equipment, and training). However, we have not found any conceptualization in the literature of what demand, capacity, or even alignment includes at other system levels or at the “blunt end” of care, nor of how top managers work in practice to align demand and capacity.

In addition to the need for research exploring the blunt end of care [3–6] and the operationalization of the systems perspective in the CARE model, recent studies highlight leadership as a central component in facilitating resilience performance and promoting safe, high-quality care. A progress report on developments in patient safety research emphasizes the need for *stronger leadership commitment,* especially in quality improvement efforts [14]. The importance of leadership at different levels in facilitating resilience has been highlighted, e.g., effective leadership in healthcare teams [8], competent frontline and/or middle managers at the unit level [11, 12, 15–17], and strong local and national leadership [18]. The study by Ree et al. [17] of managers in nursing homes and homecare services in Norway suggests several management strategies to support resilience in healthcare, including engaging staff in collaborative efforts and promoting learning environments. Other studies underline the importance of transparent, accessible, and strong leadership practices [19], empowered leadership [20], strong crisis leadership, and continuously evaluating and adapting [21]. Still, few studies seem to have specifically investigated the work conducted at the top management level, above the hospital or primary care unit level, and the efforts made to align demand and capacity. At higher levels of the system, laws, national regulations, and reforms [5, 22], as well as societal expectations, are among the demands that need to be considered, while capacity might include both private and public care providers, higher education facilities, and public health actors [5]. We thus complement previous research by providing insights into everyday work among regional top management and by that, adding an operationalization of the resilience concept by unfolding the work done to align demands and capacity at the top management level.

### Aim

The aim of this study was to explore how top managers work to align demand and capacity in a healthcare region in Sweden. The study is part of a larger project, conducted in a Swedish the region, with the overall aim to study how good leadership is practiced in healthcare.

## Methods

### Design

An ethnographic study with a qualitative approach [23] was conducted. Multiple data collection methods, e.g., fieldnotes, participant observations, conversations, and interviews were used, to explore the daily goings-on at the top management level in a Swedish healthcare region, and how work is done to align demand and capacity. The emergent research design [24] allowed for flexibility in following matters that appeared during data collection, and provided depth and authenticity, making it appropriate for this study. As the focus of this study was to investigate a specific issue, i.e. how top managers work to align demand and capacity, rather than to provide a holistic cultural analysis, the research strategy was inspired by what is called Focused Ethnography (FE) [23].

### Study setting

The study was performed in one of Sweden’s 21 healthcare regions. In Sweden, each region is self-governed and responsible for providing healthcare to the population in a specific geographic area. Excluding social services and municipal elder care, the healthcare regions are responsible for providing acute care, psychiatric care and primary healthcare for all its citizens. Healthcare in Sweden is primarily tax-financed and based on principles of equal access to quality care. The regions are also responsible for public transportation and various types of developmental work in areas like culture, innovation, and infrastructure [25]. The chosen region stands out in national comparisons for having high-quality care with a sustainable development over time. This region is of medium size and encompasses both urban and rural areas, with a relatively high proportion of elderly people.

### Data collection and participants

FE is typically conducted over a short period of time, with researchers working in the field during specific times to observe certain events, whereas fieldwork in traditional ethnography is conducted over a long period [23, 26]. One of the authors, MvK, spent a total of three months, during 2018–2021, following daily practices of healthcare in different parts and at different levels of the region. In accordance with the ethnographic approach, MvK spent considerable time getting familiar with the environment and building trust with the participants. The field visits conducted were purposeful, using specific time frames and/or events, in line with the FE approach. Also, in line with principles of FE, background knowledge informed the research question, interview guides, and questions used during conversations [23]. All data collection was performed by MvK, an experienced qualitative researcher. Included in this study are all observations, interviews and conversations that were conducted at top management level during the visits in 2018–2021.

### Observations

Observations in this study included the two types of top management team meetings with overarching responsibility for all healthcare conducted in the region: 1) the regional management team (RMT) (in the results presented as RMT01–03) consisting of the top managers for all the areas that fall under the responsibilities of the region (see above), and 2) the regional healthcare management team (RHCMT) (in the results presented as RHCMT01–05) consisting of the top managers in healthcare (hospital, primary, and psychiatric care), as well as the heads of administrations with specific relevance within healthcare. The proportion of men and women in both teams are well within the 40–60% gender balance zone. Both management teams meet once a month. No formal observation sheet was used when the observations were conducted. Fieldnotes written during the observations were used as data.

The researcher involved was introduced as a leadership researcher for the team members and mainly took the role of a non-active observer during the meetings. However, she was on a few occasions approached in her researcher role and asked to reflect on issues related to the study. These questions were postponed to separate meetings (not included in this study) where preliminary findings from the study were presented to the organization. That the degree of participation varies in observation studies is in line with the tradition of FE, where there is a continuum of different roles that the researcher can adopt during observations. These range from acting as a participant, to participant-as-observer, observer-as-participant, and observer [23].

### Interviews and conversations

Interviews and conversations held with members in the two management teams during the visits are included in this study (Int01–03 and Conv01–12). The following roles are represented in the interview and/or conversation data (4 women and 3 men): regional executive officer, healthcare director, primary healthcare director, psychiatric care director, regional director of development, human resource director, and planning director. The interviews were planned in advance, for a specific visiting period, whereas conversations took place ad hoc following casual encounters, as is common in an FE approach [23].

The interviews were semi-structured, explorative, and lasted around 1.5 h each. The interviews were conducted by MvK and took place at the top managers’ offices. The interview guide (see attachment 1) used was constructed for the larger project and organized around different themes relating to how leadership was performed through every-day work and how good work environments were created through every-day (leadership) work within the Region. During the interviews, the interviewees were encouraged to freely reflect upon different aspects of the subjects discussed. There were explorative questions related to each theme, followed by clarifying probing questions such as "Could you describe more on how this occurred…?" or "…can you elaborate more on how you handled this…?”. The interview guide was adapted to each interviewee; thus, each theme was visited to a differing degree depending on the interviewee. The interviews were recorded on an MP3 recording device and transcribed verbatim.

The conversations varied in length and no formal guide was used. Fieldnotes were written during conversations and used as data. In total, the data presented in this paper include 3 RMT meetings, 5 RHCMT meetings, 3 semi-structured interviews and 12 conversations with different members of the two management teams.

### Data analysis

**Fig. 1:**
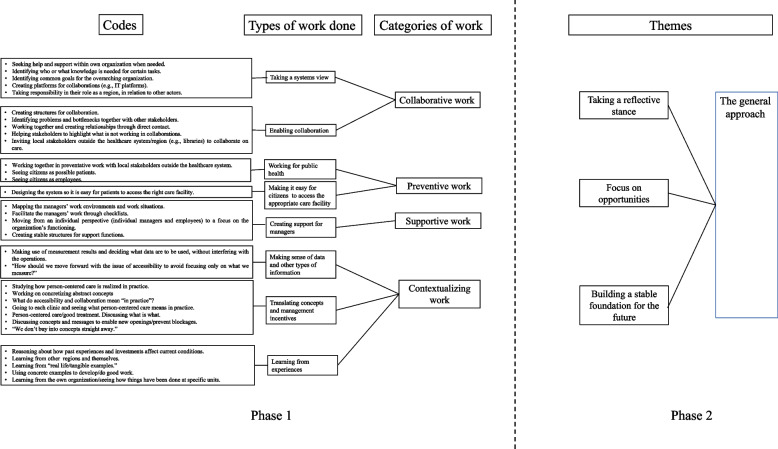
Overview of the coding structure following the two analysis phases. The figure displays the types of work conducted by top management to align demand and capacity, and how these form four categories of alignment work. The figure also displays the themes that together constitute the general approach that permeated the Region studied

The process for data analysis was based on the steps of reflexive thematic analysis (i.e.; i) familiarization with the data, ii) generating initial codes, iii) searching for themes, iv) reviewing themes, v) defining and naming themes, and vi) producing the report) [27, 28]. Initially, all collected data were read through several times by three of the authors (IS, MvK, LSA) to get familiar with data. Then, the researchers extracted and organized data that were related to either demand, capacity and/or alignment, to be able to answer the aim of the study. We theoretically based our understanding and analysis of the concepts demand, capacity, and alignment on the descriptions in the CARE model [10, 13] and its more extended version [5, 22]. Demand refers to that which generates things that need to be done and capacity refers to the resources or conditions needed to meet the demands. Alignment is the balancing between demand and capacity. The participants did not themselves use the words alignment, demand, or capacity, but some of their narratives were interpreted as referring to the meanings of these terms. This extracted data was then (inductively) coded according to types of work and grouped into categories of work. These categories of work were close to data and distinguished from the themes that were found to be present on a more abstract level (See Fig. 1).

During the work with generating the initial codes (see phase 1 Fig. 1), which revealed four types (or categories) of alignment work, it was noted that the work activities identified appeared to be conducted using a certain approach or mindset. This was evident across all extracted data and in the original dataset and resulted in the data analysis entering a second phase which focused on identifying aspects that, together with the four identified categories of work, could describe how alignment work was conducted in practice by top management in the studied region. During this phase, the entire dataset was revisited and three overarching aspects (or themes) that constituted a general approach were identified through discussions and analysis. For an overview of the coding structure, see Fig. 1.

**Fig. 2:**
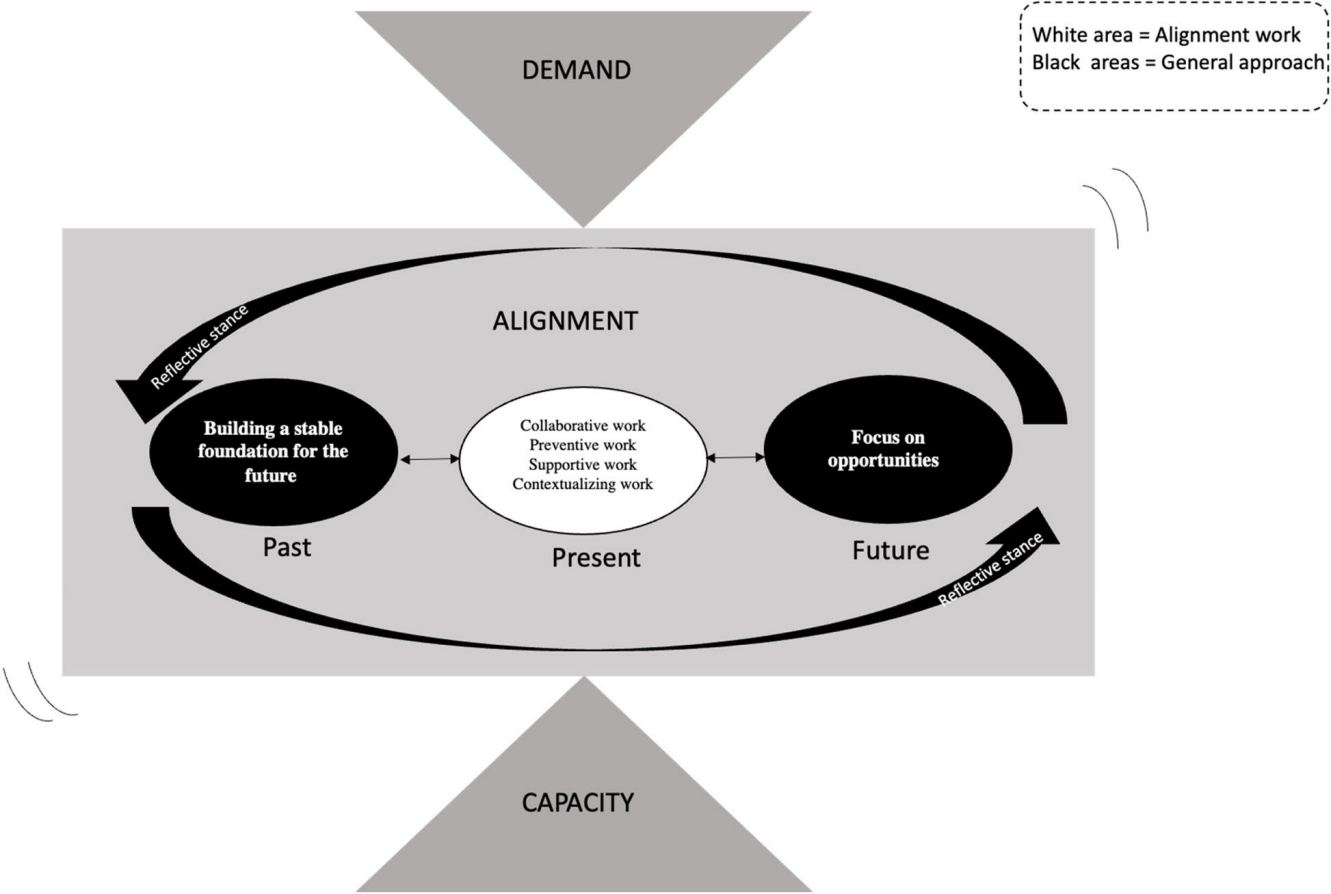
Relationships between the general approach and the categories of work constituting alignment in practice. Overview of relationships between the aspects that constitute the general approach and the work conducted to align demand and capacity. The figure illustrates how alignment is a balancing act between demand and capacity. Further, it illustrates how the four categories of work are conducted in the present, and how, through the reflective stance, these actions are connected to different points in time and how they relate to the aspects that constitute the general approach. It is illustrated that and how the work performed, and the general approach are both needed to constitute alignment in practice

Once these two phases were completed, the results were interpreted and conceptualized in relation to the entire dataset and the study as a whole, so the final report on the results could be written [27]. In Fig. 2, we display the dynamic relationships between the categories of codes and the themes visualized in the coding structure, which transformed the static data structure into a dynamic theory model [29]. To ensure internal homogeneity and external heterogeneity in the data, the categories and themes were repeatedly revisited, reviewed, and discussed by all authors.

In the results section, /…/ is used to indicate an omission, and *[…]* indicates an addition/clarification. Both are used to indicate alterations in the quote to clarify the content and/or to protect the anonymity of the informants. The overall meanings of the citations have not been altered. All quotes have been translated from Swedish.

## Results

The data showed how alignment work was done through active reflection that built on past experiences and on structures built into the organization at the same time as taking future potential outcomes and consequences into account. It was noted during interviews, conversations, and meetings that a general approach permeated the organization, which enabled connecting actions, i.e., different forms of alignment work, occurring at different points in time, and connecting different types of knowledge across organizational borders and stakeholders. As putting this approach into practice requires work in practice, we present the categories of work and the general approach separately in the result’s section, since the general approach underpins the four categories of work. We suggest that the general approach can be seen as the soil through and/or upon which collaborative work, preventive work, supportive work, and contextualizing work grow into alignment. As illustrated in Fig. 2, demand and capacity both feed into alignment, which is conceptualized as a balancing act between the two. In the following, categories of work will be presented with types of work as subcategories. Thereafter, the themes that constitute the general approach will be presented.

### Categories of alignment work

#### Collaborative work

To align demand and capacity, top management was engaged in efforts to shift perspectives, seeing all the different parts of the system, as well as the bigger picture. Two types of collaborative work were identified, i.e., taking a systems view and working to enable collaboration.

##### Taking a systems view

To align demand and capacity, top management adopted a systems view, shifting their focus from details to “the bigger picture” and trying to look beyond organizational borders and focus on “the whole.” This was done in various ways in relation to different external and internal parts of the system, employees, and patients. For example, there were times when the top management teams deemed that some issues were better handled in another part of the system. In such cases, they would discuss which people were best suited to deal with these issues and how the management team could help and support this.

In discussing a new governmental initiative, “good quality local healthcare” it was mentioned that this was an issue for the whole system, not only a particular part of it:

*It is not possible to strengthen primary care without sacrificing something else. Everyone needs to be able to raise their gaze and see the bigger picture in order for it to be possible to bring care closer to the patients.* (Conv01)

Another way of taking a systems view was in relation to the employees. When discussing a new governmental initiative, it was thoroughly discussed how it would affect the work of the employees in practice:

*“From the employees’ point of view … [there will be] too many changes and groups?”* (RHCMT02)

This systems view also encompassed adaptability to patient needs. When discussing new ways of providing care, the different needs of patients were highlighted:

*We have had very old people who have attended therapy online. But at the same time, we have those who want to meet their therapist in person. These choices differ and look different for different individuals … we need to take the perspectives of the patients into account.* (RHCMT03)

##### Enabling collaboration

To align demand and capacity, the management teams needed knowledge and input from different parts of the healthcare system and for these parts to “work together.” This was achieved through active work in creating good relationships between stakeholders within and outside the healthcare system. In practice, creating and maintaining such relationships entailed not being afraid of “picking up the phone” and call another stakeholder, both to manage difficulties and to praise each other. Revealing how the different actors in the healthcare system worked “together,” one of the interviewees said:

*I mean when hospital X received an award /…/ then* [the manager of the hospital] *called the surrounding municipalities. And told them: “Well, you helped us* [to get this award] *because you made sure we have the right patients at our hospital …”* (Int01)

Another way to enable collaboration was trying to identify and align demand and capacity in the entire region, regardless of organizational borders. One example of this was identifying critical gaps between different organizations. Many different types of care providers and stakeholders operated in the studied healthcare region. Sometimes, it was not always clear which unit or organization could help a specific patient in the best way and how different stakeholders could collaborate. To help stakeholders overcome collaborative difficulties, meetings were organized and facilitated by top management, at which specific cases were brought up and discussed. One of the interviewees described this as follows:

*So we looked at specific cases. The school services identified cases and psychiatric services identified cases, healthcare, like hospital care and social services identified cases, patient cases or student cases where the collaboration had failed, or cases where it had worked well. And then we anonymized them and then we discussed them in groups together.* (Int02)

In this way, top management took responsibility for enabling collaboration – not leaving this entirely up to the managers at lower levels of the healthcare system. By studying and analyzing the capacities and demands of different stakeholders, they aimed to increase the region’s capacity to provide good care.

#### Preventive work

This category of work included ways of working preventively; to make sure patients end up in the right care facility and/or working with public health to prevent inhabitants from being patients in the future.

##### Working for public health

One way to align demand and capacity was related to the management of potential demands. Here top management conducted work activities aimed at preventing inhabitants future need of healthcare. One such effort involved investigating how various local stakeholders could contribute to health; for example, local libraries were asked how they could contribute to public health. In line with this, a general citizens perspective permeated the organization. Another example of this was a discussion at one of the management team meetings (RMT02) on how to promote public health among the inhabitants in the region. Here the focus of the discussion was the region’s own employees, since the region itself is a big employer. Supporting good health and good working environments for the employees in the Region was also seen as a tool to work preventively with public health as these employees were also seen as citizens. The discussion resulted in suggestions on how to work preventively through attracting and retaining personnel through supporting a good working environment.

##### Making it easy for citizens to access the appropriate care facility

In order to make the best use of the capacity available, given current demands, it was mentioned how the region needed to organize its services in a way that the inhabitants in the geographical area would find easy to navigate. In one of the regional health care managements meetings it was formulated like this:

*… we have been too focused on our own ways of organizing things … it’s not the patients’ fault if they can’t find the way!* (RHCMT01)

This was also mentioned at another RHCMT meeting:

*We really need to drop the idea that it’s the patient who goes to the wrong place. It’s not the patient who gets it wrong, we’re the ones who create the system.* (RHCMT04)

One of the interviewees expressed a similar view:

*A department store would never blame the customers for going in the wrong direction. Obviously, we haven’t designed the healthcare system in an appropriate way that is adapted to suit patients’ care needs.* (Int01)

#### Supportive work

This category of work represents work to support managers, on different levels of the health care system, so that they in turn can support their coworkers. This involved for example providing good working conditions and support for the managers on all levels in the Region.

##### Creating support for managers

Top management highlighted that the managers needed support and the right prerequisites, including administrative support, to be able to support their employees in their clinical work. The quotes below illustrate how top management aimed to support managers throughout the organization and help them identify the capacities needed to meet organizational demands.

During meetings and discussions, it was a strong focus on creating good work environment in the Region and that creating support structures for this was an organizational responsibility. For example, a survey on staff wellbeing and the work environment was distributed to all employees on a regular basis. At an RMT meeting, the managers discussed how the results from these surveys should be used as indicators of how the organization was functioning, rather than focusing on individuals’ performance:

*We should try to drop the idea that the survey is an evaluation of the manager, it’s about the organizational level …It shouldn’t be a beauty pageant for the managers…* (RMT01)

The same went for the employees:

*We should never see an issue as connected only to any one individual …* (RMT01)

During interviews and meetings, it was articulated that it should be “easy” to be a manager within the region, at all levels; to make it easy one interviewee explains how:

*… we have /… / created checklists and agendas and made suggestions to avoid losing track of [important] aspects /… /. The work environment should be a top priority in all contexts and so on. We have worked a lot with these things.* (Int03)

#### Contextualizing work

Here, work centered around managing the risk of just accepting indicators, initiatives, and trends, rather than putting them into their contexts. This work was seen as a way to align demand and capacity.

##### Making sense of data and other types of information

To manage future demands, gain insight into current capacities, and align the two, various types of measurements were used within the region. These were discussed at some length, as the ambition was to use the data generated in an appropriate way. The potential difficulties of using data to manage future demands were also discussed:

*“Isn’t there a risk that we measure what we already do? What we want to do is make a real shift, right?”* (RHCMT01)

Another discussion focused on the differences between “soft” and “hard” data, and how to manage these two to increase knowledge on current capacities and thereby better align demands and capacities. More specifically, the issue of care accessibility was discussed during a meeting:

*“How should we move forward with the issue of accessibility … to avoid focusing only on what we measure … we need to have another discussion … we need to understand how care accessibility is experienced.”* (RHCMT01)

One aspect that tied into how the Region reasoned about and managed data, was that they also created their own demands, which went beyond what was actually required of the region. One example of this concerned the patient experience of coordination: it was stated that this was an important indicator of good care that should be followed and measured and included in the regional plan on healthcare provision, which it was not at the time. In other words, data alone were not seen as enough to manage future demands. Rather, what types of data were gathered and how they were used were important aspects for facilitating learning and adaption in the organization.

##### Translating concepts and management incentives

To find a resilient way of aligning demand and capacity, there was a clear ambition to discuss how new demands, government initiatives, and management trends in healthcare would impact the region and to contextualize them before deciding how to implement them (if at all). One common stance in the studied region was:

“We don’t buy into new concepts straight away.” (Conv01)

This was also mentioned during a meeting with the RHCMT:

*… Maybe we need to start talking about what they* [the concepts] *mean … we need to take it one clinic at a time /…/ “what does this mean for you” … go to every clinic: “what could work for you? What does this look like at your place?”* (RHCMT03)

##### Learning from experiences

During meetings, participants considered and discussed how past experiences from their region could be used for future learnings, and also how to learn from other healthcare regions. One example could be using best practices and models developed elsewhere (RMT01). During one meeting, the following questions were posed by a member of the RHCMT, illustrating how top management aimed to relate their own past experiences to current research, to enhance learning:

*What did we learn from the “cancer implementation”? What does research tell us? Should a pilot be used or not?* (RHCMT01)

### The general approach in the Region

Three aspects were identified that together made up a general approach that permeated the entire region.

#### Focus on opportunities

Overall, a tendency to focus on the future and opportunities rather than on obstacles was seen in the data. Obviously, some obstacles existed and were mentioned, but these were put on a back burner, in favor of focusing on possibilities. For example, rather than dwelling on all the things that complicated collaboration, the focus was on what could be done despite the obstacles:

… all these organizational constraints that actually say you can't do this and you can't do that. You have different electronic medical record systems, and you have different laws that … Take for instance the social services – those laws versus the healthcare laws are not always compatible. But if you ignore what doesn't work and try to find what does, it's much easier. (Int02)

#### Building a stable foundation for the future

There was also a focus on “building on the past” – to learn from experiences and gain cohesion throughout the healthcare system:

*… I mean, we're a big organization /…/ we have retained our ways of working /…/ always keeping an open dialogue /…/ There is a clear connection from the overall document describing our budget and plan, which is also a political document describing what, what we should do … /…/ And based on that, we operationalize it in all our central organizations /…/ … that creates cohesion.* (Int01)

This way of building on previous experience was evident in the way the region was “working together.” This setup had been used within the region for a long time, and having an established way for stakeholders to collaborate within the healthcare system enabled smoother adaptation to new laws and regulations. For example, one interviewee said:

*And now, when we are trying to change the way we work with regards to the new law on collaboration in discharge, which was introduced in January 2018 /…/ you clearly see the benefits of having an established platform for collaboration. We have had this collaboration* [for several years]*. It’s made it much easier to agree on how we* [different types of actors] *can do this together.* (Int02)

#### Taking a reflective stance

During both RHCMT and RMT meetings, considerable time was regularly set aside for free reflection, during which participants were encouraged to freely discuss. These discussions showed how the possibility to be reflective was founded on trust between the meeting participants and the idea that everyone’s opinion was equally important. The reflections usually focused on a certain issue on the agenda, but themes varied from time to time. Multiple aspects contributed to the reflections, with participants helping each other to shift perspective from details to seeing the bigger picture. Together, they kept a focus on the overall purpose of providing good care to the region’s citizens. It was noted how the perspectives of both employees, patients, and other stakeholders were raised during these reflections.

This reflective stance was also reflected in how the organization was described in a conversation with a management team member. According to that person, the region should be able to adapt to trends that “we do not even know about yet.” The management team member likened the region to a creature that could adapt in different ways: “walking around, lying flat, and climbing over things if needed”. The main point was that not all new trends should be implemented in the region, according to this participant.

*“The region needs to have the capacity for learning and for reflection, to know when to adopt new trends and when to say no.”* (Conv12)

## Discussion

This study explored top managers’ work to align demand and capacity in a healthcare region in Sweden. The results show how alignment is understood as a balancing act between demand and capacity that requires substantial work in practice. Four categories of alignment work were identified, all conducted in the present (collaborative work, preventive work, supportive work, and contextualizing work). In tandem with the general approach (taking a reflective stance, focusing on opportunities, and building on the past), the four categories of work described created alignment in practice in the studied region.

### Alignment in practice from the top management perspective

Previous studies have provided examples of what leaders/managers *should be doing* (i.e., being present at frontline, accepting feedback from staff, considering the situation at the frontline when formulating policy) [12], our study complements this research by investigating what top management is *actually doing* in practice. While leadership, according to Lyng et al., [12] influences all (resilience) capacities, leadership is not the sole foundation of these capacities. They are all interrelated, and a lack of leadership can be compensated by strong organizational abilities [12]. Rather than merely labelling the work of top management as “leadership,” we have unfolded what the leaders actually do, when they perform this leadership. We have shown how alignment requires extensive work on the part of top management, which in this case included working to create the general approach found to permeate the organization. Moreover; most research on resilience in practice has focused on the work of frontline staff and unit managers [4, 10, 30], but it has previously been shown that the work of leaders at multiple organizational levels (including top management) [11, 16] is needed for organizations to be resilient. However, as these studies had problems identified by middle managers as their starting points for investigation, we argue that they might have missed what happens further away from the bedside and what types of activities top management engages in.

### Demand and capacity

Expanding the examples of demand and capacity given in the CARE model [10], we applied a more abstracted view of what might constitute demand and capacity, to better reflect the data at the overarching regional level. For example, we noted how top management included regulatory and societal demands (e.g., mandated regional healthcare provision, national guidelines on person-centered care, regionally set standards of care) in addition to organization-specific demands. Capacity was seen as encompassing the entire system in the region, including resources and capacity emerging from the combination of organizations and collaborations between different units and entities. When we unfolded alignment by separating work activities and the general approach, it also became evident how the region reasoned about its own demands and capacities. The participants did not dwell on either of them, focusing on opportunities rather than constraints. Further, the region developed its own additional demands, sprung from ideals and values, that sometimes corresponded to higher ambitions than the ones imposed by statutory and other types of requirements.

### Collaborative, preventive, supportive, and contextualizing work and the general approach

Looking from the perspective of top management, we were able to explore how alignment work, through the general approach, aimed at creating a resilient health care system in the Region. Sometimes, the acts of top management did not end up at the bedside. For example, one aim of collaboration with different stakeholders was to help minimize the need of healthcare for the inhabitants in the region. Similarly, the aim of some of the preventive work was to avoid having patients end up at the wrong care facility.

The categories of work conducted, were in line with what has been found at other system levels. For example, Ree et al. [17] studied managers in primary care and found that *“engaging people in collaborative and coordinated processes that adapt, enhance or reorganize system functioning, promoting possibilities for learning, growth, development and recovery of the health care system…”* This is in line with the work described in our study. What our study also showed was the simultaneous existence of a general approach that permeated the organization, which we refer to as the soil through and/or upon which collaborative work, preventive work, supportive work, and conceptualizing work grow into alignment (Fig. 2).

Previous studies have shown that organizational issues can be perceived differently at different organizational levels [11]. This means that aligning demand and capacity requires top management to keep the “system” in mind, presented here, foremost, as "taking a systems view" and in the reflective stance. The dataset also revealed that the categories of work presented, guided by the general approach, made it possible for top management to retain a focus on “the whole.” This includes how patients are viewed in the region. Rather than merely talking about patients, top management often referred to “citizens.” These were both potential patients and potential employees. Further, both people who were well and those who were not were included in the work of top management.

As noted above, at the top management level, being interested in the experience of care means taking a different stance on demands and capacities than that commonly shown in research on resilient healthcare systems. Not only healthcare facilities were included in the management view of “the whole” system, but also other types of organizations impacting on the well-being of citizens, such as libraries and regional transportation systems. By taking the perspective of top management, we got the opportunity to explore how resilience activities focused on aligning demands and capacities were spread across the whole system. The possibility and the responsibility of aligning demands with capacities, for the benefit of” the whole,” is greater for top management in the healthcare system, while middle managers and first-line managers often feel squeezed in their position between different levels of care [31–33]. Our study showed how top management in the Region used this possibility in contributing towards a resilient organization.

### Methodological considerations

To achieve trustworthiness in this study, the concepts of credibility, transferability, and dependability were considered [34]. In order to enhance credibility, top managers with different roles were included, with the aim of capturing a variety of experiences of how the participants aligned demand and capacity in their work.

An ethnographic approach allows for obtaining data with high credibility regarding how work is actually done. A triangulation of multiple data collection methods, in line with the ethnographic approach, allows different perspectives on specific issues to be disclosed and enhances the quality of the conclusions drawn.

The study was part of a larger project at different levels of healthcare in the region in 2018–2021 and all interviews and conversations that were conducted at top management level during that period are included in this study. All participants in interviews and conversations had extensive experience of top management work, enhancing credible interpretation of the data. The procedure to select participants for interviews and conversations in the study built on availability at a specific visit, or ad hoc encounters during the visits. The data were collected in a specific region with a population with certain demographic characteristics – a region that stands out in national comparisons for having high-quality care. These conditions may have affected the transferability of the results. One limitation related to the interviews and informal conversations could be that top managers might have shared information they believe would give the researchers a positive view of them in comparison with other similar organizations. Including top management from collaborating organizations would have nuanced the results and added another angle. However, this was outside the scope of the present study.

To enhance dependability and consistency in data collection over the long time period, only one of the researchers (MvK) was involved in data collection. To alleviate the possible risk of bias in participant observations, i.e., that the observer becomes allied with the population observed and loses the critical gaze, the entire research team was involved in every step in the analysis process, discussing the interpretation of codes and the themes until consensus was reached. In this way, the risk of a single researcher’s interpretation coloring the data was reduced. The methods and findings have been described and verified with representative quotations, to help the reader determine whether the findings can be transferred to other groups or contexts. Future research studies could be designed to investigate correlations between the work of top management and quality of care.

## Conclusions

This study aimed to explore how top managers work to align demand and capacity in a healthcare region in Sweden. It was shown how four categories of work; collaborative, preventive, supportive and contextualization work, together with a general approach; focusing on opportunities, building on a stable past, and taking a reflective stance, constitute alignment in practice. More; the alignment work was done in the here and now, with both the past and future in mind.

The ability to take action to benefit the whole is a possibility and a responsibility for top management. In the region studied, this was done by aligning demands with capacity based on past experiences and focusing on the available opportunities to connect knowledge needed within and across organizational boarders.

## Supplementary Information

**Additional file 1.**

## Data Availability

The data collected and analyzed in this manuscript are not publicly available due to participants not having consented to public availability. Aggregated data in Swedish are available from the corresponding author on reasonable request.

## Abbreviations

CARE model: Concepts for Applying Resilience Engineering
FE: Focused Ethnography
RHCMT: Regional healthcare management team
RMT: Regional management team
