# Supplementary material for: Unfolding alignment – How top management work to align demand and capacity: an ethnographic study of resilience in a Swedish healthcare region
Source: BMC Health Serv Res. 2023 Mar 31;23:321. doi: 10.1186/s12913-023-09291-0 (PMC10067293; doi:10.1186/s12913-023-09291-0)
Supplement: Supplementary file 1 — Additional file 1. [file 12913_2023_9291_MOESM1_ESM.pdf]

Thematic interview guide for top management

The overall purpose of the interview is to explore how leadership is exercised in everyday work in healthcare in order to contribute to a good working environment and good care. The interviewees are encouraged to freely reflect on any aspects of the subject that they find relevant.

The following themes will specifically be touched upon in the interviews with top management. Examples of formulation of questions within each theme are given.

1. **Leadership:** View of leadership in general, how the view of leadership spreads and permeates the organization, the relevance/importance of leadership for work environment/quality of care.

E.g. What does good leadership mean to you? How do you view the connection between leadership, work environment and quality of care?

2. **Person-centered care:** View of good care/person-centered care, view of the relevance/importance of organizational conditions for good care/person-centred care.

E.g. What does person-centered care mean to you? What do you see as important for healthcare as a whole to be able to provide good treatment and care to patients? How do you regard the link between leadership and quality in healthcare?

3. **Social and organizational work environment:** View of the importance of the work environment, organizational support, trust between managers and employees, employeeship

E.g. How do you see the connection between leadership and work environment? Between a good working environment and good care? How would you describe the purpose of the organization and how can you contribute to it? What makes you to feel energy and meaningfulness at work?
